# A new species of *Agaporomorphus* Zimmermann, 1921 from Peru (Coleoptera, Dytiscidae, Copelatinae)

**DOI:** 10.3897/zookeys.512.9505

**Published:** 2015-07-06

**Authors:** Lars Hendrich, Rico Apenborn, Ernst-Gerhard Burmeister, Michael Balke

**Affiliations:** 1 SNSB-Zoologische Staatssammlung, Münchhausenstrasse 21, D-81247 München, Germany; 2 Hochschule Zittau/Görlitz, Germany; 3 GeoBioCenter, Ludwig-Maximilians-University, Munich, Germany

**Keywords:** Dytiscidae, *Agaporomorphus*, *Hydrodytes*, new species, new records, habitat, Peru, Panguana, Neotropical region

## Abstract

*Agaporomorphus julianeae* **sp. n.** is described from the Biological Field Station Panguana, in Huànuco province of central Peru. The new species belongs to the *Agaporomorphus knischi*-group sensu Miller 2005. Together with *Agaporomorphus knischi* Zimmermann, 1921 and *Agaporomorphus colberti* Miller & Wheeler, 2008 this is the third species of the genus with broadly enlarged male antennomeres. The new species can be separated from *Agaporomorphus colberti* and *Agaporomorphus knischi* by the smaller please expanded male antennomere VIII, and the form of the median lobe. Important species characters (median lobe, male antennae, metafemur, colour pattern) of the new species and *Agaporomorphus knischi* are figured, and the habitat, a temporary blackwater forest pond, and its species rich water beetle coenosis are illustrated and described in detail. The Brazilian *Agaporomorphus mecolobus* Miller, 2001, only known from the type material from Sao Paulo, is here recorded for Minas Gerais. Habitus photos of four additional *Agaporomorphus* species and *Hydrodytes opalinus* (Zimmermann, 1921) are provided. Altogether ten species of *Agaporomorphus* are now known.

## Introduction

The diving beetle genus *Agaporomorphus* Zimmermann, 1921 is restricted to the Neotropical region and distributed from central Peru north to Suriname and Venezuela, and south to south-eastern Brazil and northern Argentina (Miller and Wheeler 2008, Libonatti et al. 2011). The genus was taxonomically reviewed by Miller (2001, 2005, 2014), and Miller and Wheeler (2008), who described seven of the nine known species (Nilsson 2015). Phylogenetic analyses of Copelatinae suggest a possible sister group relation of *Agaporomorphus* with the Malagasy *Madaglymbus* (Bilton et al. 2015).

Most *Agaporomorphus* are known from Brazil, Peru and Venezuela (Miller 2014). The new species of the *Agaporomorphus knischi*-group sensu Miller 2005 described herein is the fifth Peruvian species and the tenth species of *Agaporomorphus* to date. The biology of *Agaporomorphus* has remained virtually unknown. Almost all specimens were collected with different light traps (Miller 2001, 2005, Miller and Wheeler 2008) except for *Agaporomorphus sharynae* Miller, 2014 which was collected among accumulations of fallen leaves in a small backwater of a sandy and slow flowing forest stream in Venezuela (Miller 2014).

The research station Panguana, in the Peruvian lowland rainforest in Province Huànuco of central Peru, is situated close to the western slopes of the Andes on the southern bank of the Rio Llullapichis (= Yuyapichis) an eastern affluent of the Rio Pachitea, some 170 km south of the town of Pucallpa. The station was founded by the German zoologist couple Maria Koepcke and Hans-Wilhelm Koepcke in 1968 and is now operated by Juliane Diller and the Zoologische Staatssammlung in Munich, Germany (Schlüter 2005).

During eight field trips (1982–2013) E.-G. Burmeister spent several months collecting thousands of aquatic insects, including the first specimens of the new *Agaporomorphus* described herein. In 2013 R. Apenborn travelled to Panguana for eight weeks collecting aquatic beetles including additional specimens of *Agaporomorphus* and the first *Hydrodytes* known from that area. In an unpublished report Apenborn (2013) gave a first overview about the water beetle fauna of Panguana, listing 122 species in ten families.

Besides the description of the tenth species of the genus we present a detailed description of an *Agaporomorphus* habitat, which housed a stable population for many years, and its species rich water beetle coenosis. Furthermore, habitus photos of four additional *Agaporomorphus* species and *Hydrodytes opalinus* (Zimmermann, 1921) are provided for the first time.

## Material and methods

Beetles were studied with a Leica MZ 12.5 microscope at 10–100×. Habitus photographs were taken with a digital photo imaging system, composed of a Leica Z 6 APO and a Nikon V1 camera. The genitalia images were produced with a Mitutoyo M Plan Apo ELWD lens attached via a bellows to a Nikon D3 camera. Image stacks were produced by moving the camera with a StackShot macrorail. Image stacks were aligned and assembled with the computer software Helicon Focus 4.77TM.

Label data of type material are cited in quotation marks. All type specimens of the herein described species are provided with red labels. The terminology to denote the orientation of the genitalia follows Miller and Nilsson (2003). The following abbreviations were used: TL (total length), TL-H (total length without head), and MW (maximum width). Exact label data are cited for the type material. Additional remarks are found in square brackets.

We used Google Earth (http://earth.google.com) to locate localities and the coordinates are given in decimal notation. Our map bases on “MICROSOFT ENCARTA World-Atlas 2000”.

The specimens included in this study are deposited in the following collections:

MUSM Museo de Historia Natural, Universidad Nacional Mayor de San Marcos, Lima, Peru

MNHN Muséum national d´Histoire naturelle, Paris, France

NMPC Národní muzeum, Praha, Czech Republic

SMTD Staatliches Museum für Tierkunde Dresden, Germany

UFMT Universidade Federal de Mato Grosso, Brazil

ZSM Zoologische Staatssammlung, München, Germany

The descriptive style follows Miller (2014).

### Checklist of *Agaporomorphus*

*Agaporomorphus colberti* Miller & Wheeler, 2008 Venezuela

*Agaporomorphus dolichodactylus* Miller, 2001 Brazil, Bolivia

*Agaporomorphus grandisinuatus* Miller, 2001 Brazil, Peru

*Agaporomorphus julianeae* sp. n. Peru

*Agaporomorphus knischi* Zimmermann, 1921 Brazil, Peru, Bolivia

*Agaporomorphus mecolobus* Miller, 2001 Brazil

*Agaporomorphus pereirai* Guignot, 1957 Brazil, Surinam

*Agaporomorphus sharynae* Miller, 2014 Venezuela

*Agaporomorphus silvaticus* Miller, 2005 Peru

*Agaporomorphus tambopatensis* Miller, 2005 Peru

## Taxonomy and faunistics

### Agaporomorphus julianeae sp. n.

Taxon classificationAnimaliaColeopteraDytiscidae

http://zoobank.org/50D5990E-3D52-4592-82AA-B06D7B885E2B

Figs 1 , 3 , 6 , 8 , 10 , 11 , 12–14

#### Type locality.

Peru, Huànuco province, Rio Yuyapichis, Biological Field Station Panguana, 260 m [9°37'S, 74°56'W], temporary forest pond.

#### Type material.

**Holotype** ♂: “Peru, Prov. Huànuco, Rio Yuyapichis, Biol. Stat. Panguana östl. Ort, 9°37'S, 74°56'W 6–17. April 2003 leg. H.J. u. E.-G. Burmeister”; “HOLOTYPE Agaporomorphus julianeae sp. nov. Hendrich, Apenborn, Balke & Burmeister des. 2013 [red label, printed]” (MUSM). **Paratypes**: 2 ♂♂ 5 ♀♀, same label data as holotype (ZSM); 3 ♂♂ 8 ♀♀, “Peru, Dept. Huànuco, ACP Panguana, Rio Yuyapichis, östl. Ort, 9°37'S, 74°56'W, 230m, 10.05.–25.7.2013, leg. R. Apenborn” (NMPC, ZSM). Each paratype is provided with the respective red printed label.

#### Description of male holotype.

Measurements. Holotype: TL = 3.5 mm, TL-H = 3.2 mm, MW = 1.65 mm. Paratypes: TL = 3.3–3.5 mm, TL-H = 3.0–3.2 mm, MW = 1.6–1.7 mm.

**Figure 1.**
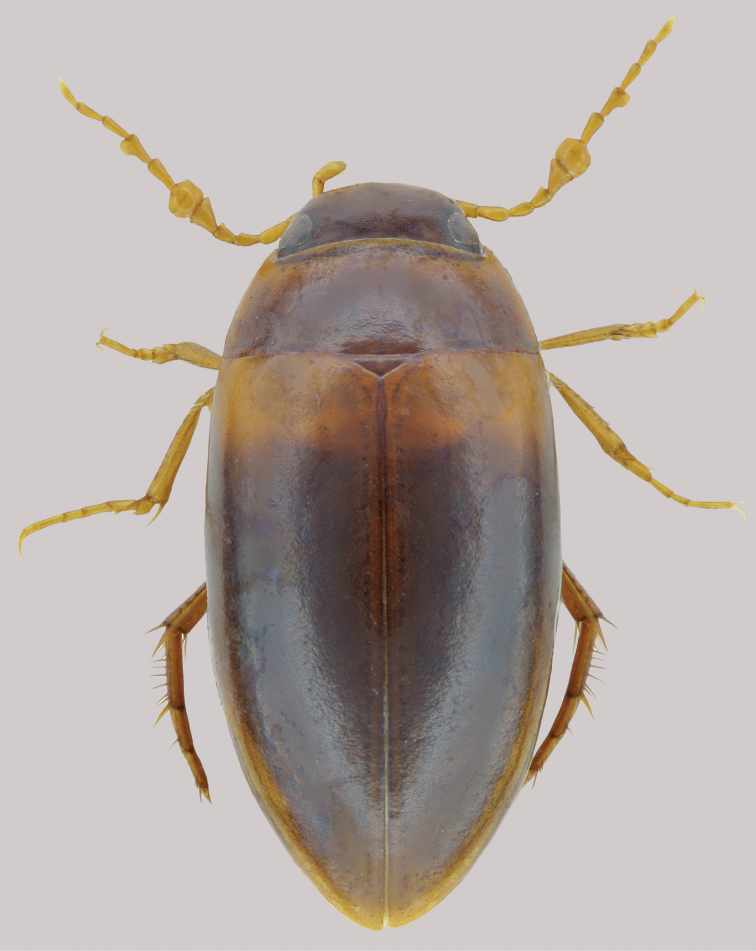
Habitus of *Agaporomorphus julianeae* sp. n., paratype, male, 3.5 mm.

**Coloration** (Fig. 1). **Head** yellowish-brown to brown. Pronotum yellowish-brown medially and lighter laterally. Elytra with most of surface yellowish-brown to brown, with broad, yellow basal band. Ventral surfaces and appendages yellow except abdominal ventrites yellowish-brown.

**Sculpture and structure.** Head and pronotum with microreticulation consisting of fine cells, with few very fine punctures interspersed; pronotum with narrow lateral pronotal margin. Prosternum medially strongly carinate, carina extending onto prosternal process; prosternal process medially with a rounded longitudinal carina extending to apex, laterally with strongly beaded margins, apex pointed. Elytron covered with extremely fine, evenly spaced, short striae, striae more punctiform laterally and apically. Metafemur moderately broad, length about 2.8 × greatest width (Fig. 8). Metacoxae smooth, impunctate; metacoxal lines closely approximated.

**Figures 2–5.**
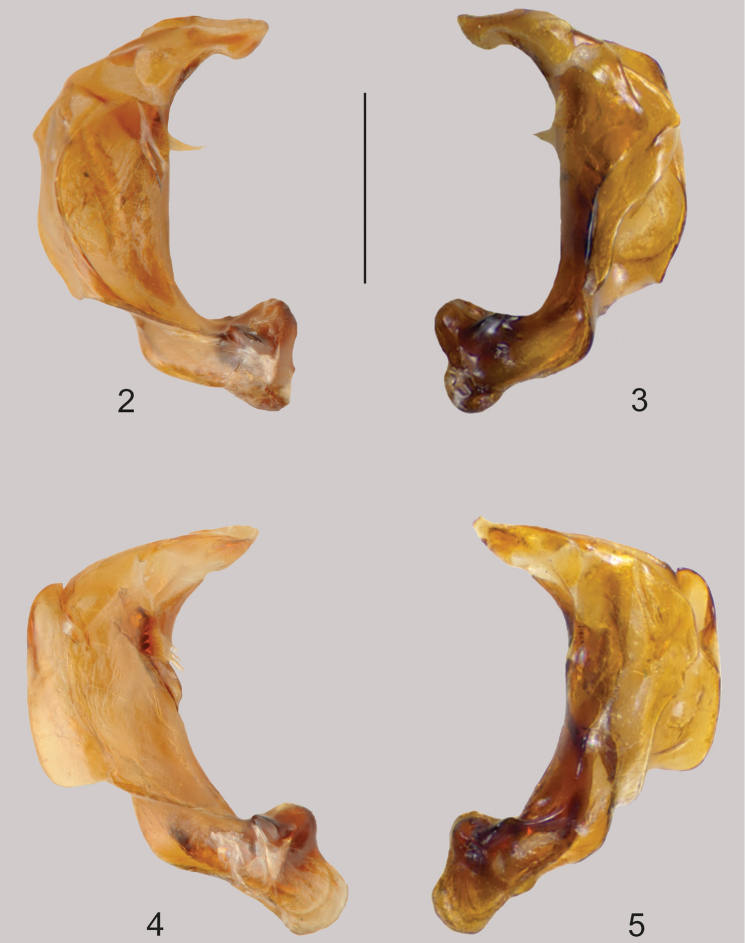
Aedeagus of *Agaporomorphus julianeae* sp. n., **2–3** median lobe in lateral view, right and left side; *Agaporomorphus knischi* **4–5** median lobe in lateral view, right and left side. Scale bar = 0.4 mm.

**Male genitalia.** Median lobe in lateral aspect robust and strongly curved medially; apex elongate, with distinct dorsally-directed lobe on right side medially and very broad, angular region sub-basally, with linear series of fine setae on each side of dorsal midline (Figs 2, 3). Parameres broad, strongly curved, apex strongly curved, with series of long setae medially along internal membrane.

**Figures 6–9.**
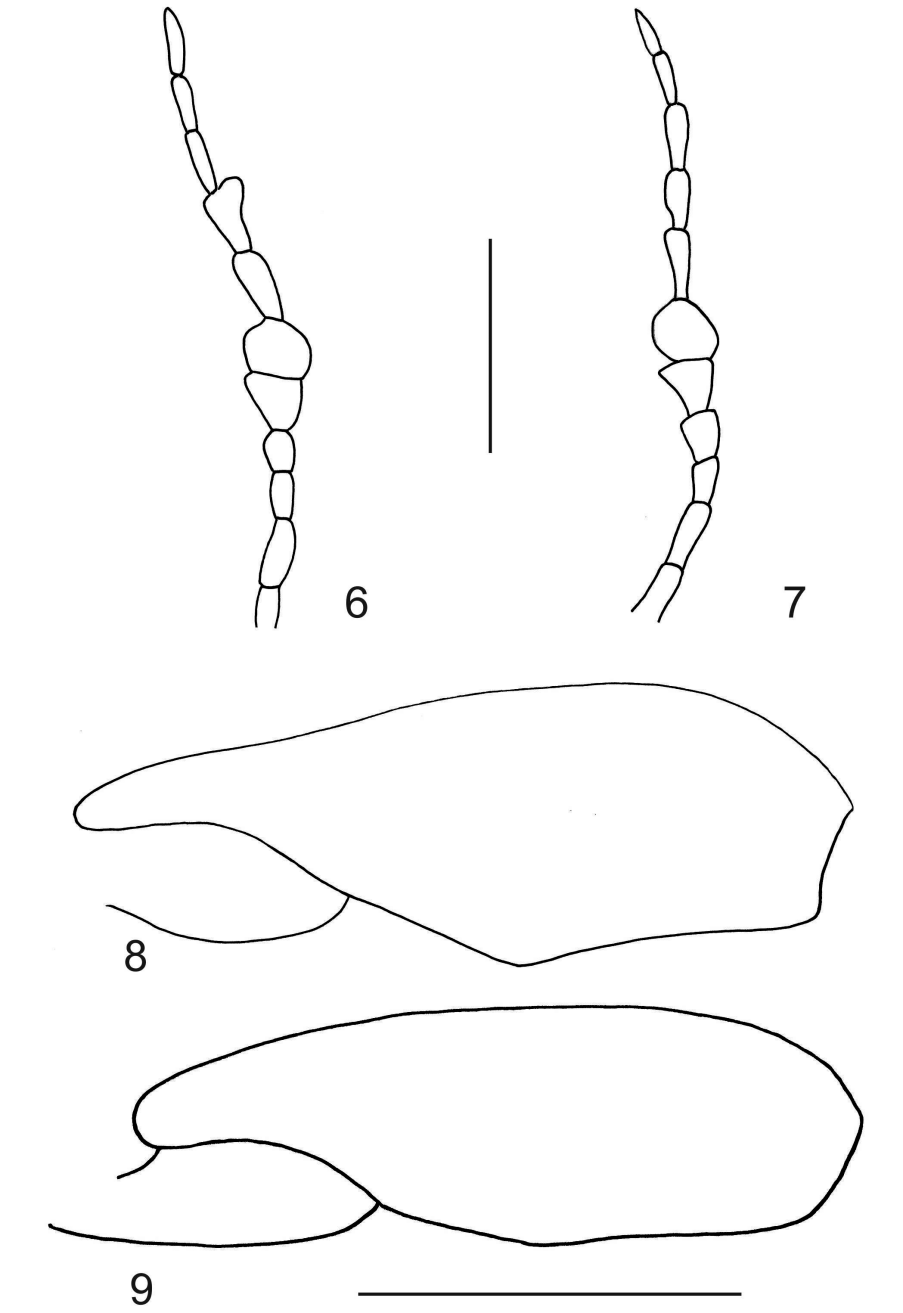
Right male antennae of *Agaporomorphus julianeae* sp. n. (**6**) and *Agaporomorphus knischi* (**7**); right metatrochanter and metafemur, anterior aspect of *Agaporomorphus julianeae* sp. n. (**8**) and *Agaporomorphus knischi* (**9**). Scale bars = 0.5 mm.

**Sexual dimorphism.** Male protarsal claws unmodified; pro- and mesotarsal claws about half length of mesotarsomere V; without apical lobe on mesotarsomere V; protarsomeres I and II broadened, protarsomere I with two large adhesive setae, protarsomere II without adhesive setae; mesotarsomeres I and II slightly broadened, mesotarsomere I with one large, medial adhesive seta and two large, apical adhesive setae, mesotarsomere II with two moderately sized apical sucker disks. Male with small but distinct triangular, posteriorly-directed tooth-like prominence medially along posterior margin of visible abdominal ventrite V and with broad and shallow depression medially on abdominal ventrite VI. Male with vague parallel series of rugulosities on each side of midline on abdominal ventrite III. Antennomeres V, VI and VIII modified; V broadly triangular, VIII broad with large posterior emargination (as in Fig. 6). Pro- and mesotarsomeres of female unmodified. Shallow depression medially on abdominal ventrite VI and parallel series of rugulosities on each side of midline on abdominal ventrite III absent. Antennomeres and femur of female unmodified.

**Measurements.** TL = 3.3–3.5 mm, TL-H = 3.0–3.2 mm, MW = 1.6–1.7 mm.

#### Etymology.

The new species is named after Juliane Diller, deputy director of the Zoologische Staatssammlung in Munich, and head and owner of the Biological Field Station Panguna, in recognition of her longstanding efforts in biological research and nature conservation in Peru.

#### Affinities.

The new species can be clearly placed in the *Agaporomorphus knischi* species-group sensu Miller 2005, characterized by distinctly modified male genitalia and expanded male antennomeres. Within this group, *Agaporomorphus julianeae* sp. n. is most similar to *Agaporomorphus knischi*, but differs from that species in the shape of the median lobe (Figs 2–5), expanded male antennomere VIII (Figs 6, 7) and different form of the metafemur (Figs 8, 9). Furthermore, the posteromedial triangular spine on abdominal ventrite V is slightly larger in *Agaporomorphus julianeae* sp. n. than in *Agaporomorphus knischi* (see Miller 2001, Fig. 32).

#### Distribution.

**Figure 10.**
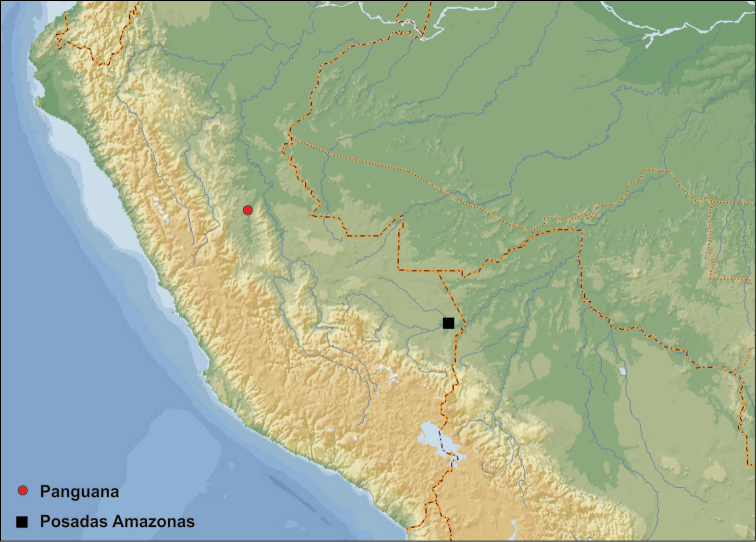
Records of *Agaporomorphus* species in Peru. *Agaporomorphus julianeae* sp. n. and *Agaporomorphus tambopatensis* (Panguana, red dot); *Agaporomorphus grandisinuatus*, *Agaporomorphus knischi*, *Agaporomorphus silvaticus* and *Agaporomorphus tambopatensis* (Posadas Amazonas, black square).

Only known from the type locality in Panguana, Peru. The occurrence in other parts of Peru is likely (Fig. 10).

#### Habitat.

**Figure 11.**
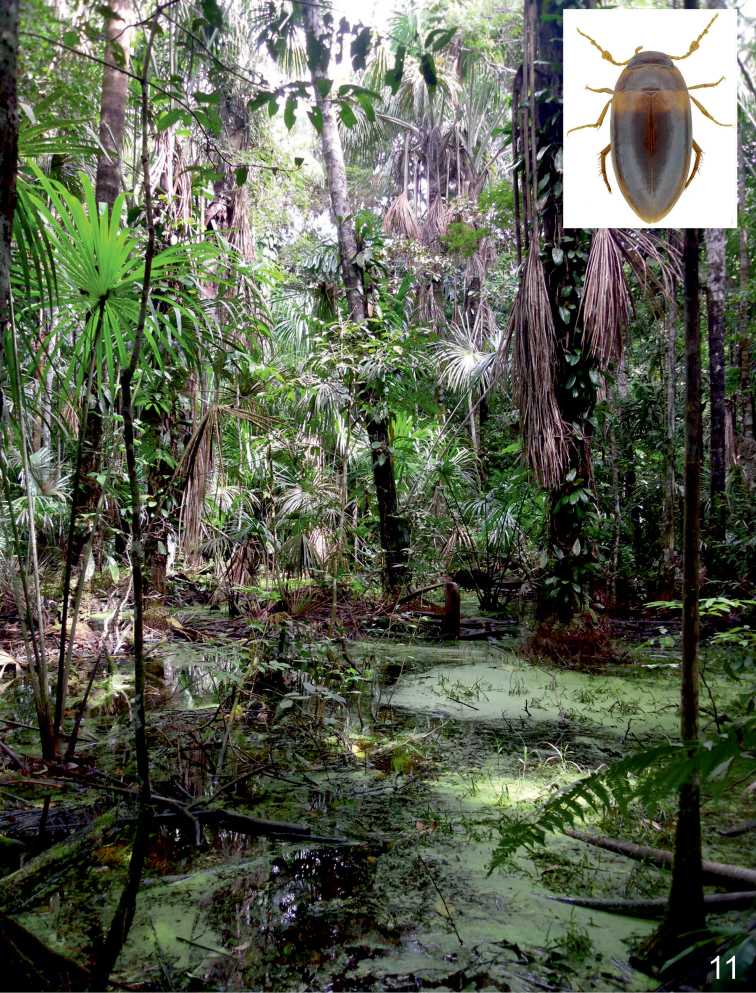
Biological Field Station Panguana, Huànuco province of central Peru: Aguajal forest pond, habitat of *Agaporomorphus julianeae* sp. n.

**Figures 12–14.**
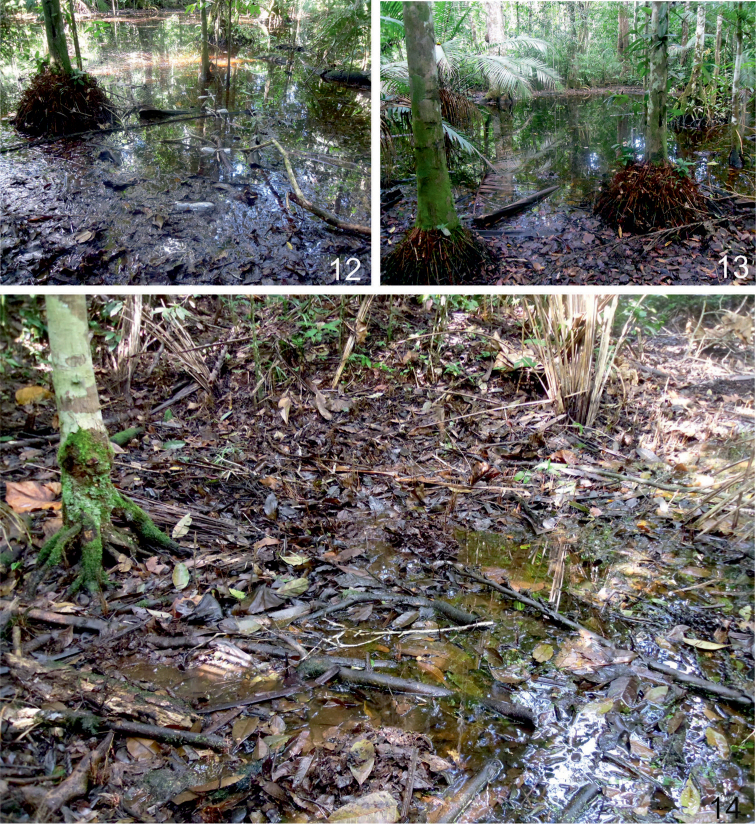
Biological Field Station Panguana, Huànuco province of central Peru: Estanque forest pond (**12, 13**), Shallow puddles and accumulations of fallen wet leaves at the edge of Aguajal (**14**), habitat of *Agaporomorphus julianeae* sp. n. and *Agaporomorphus tambopatensis*, and *Hydrodytes opalinus*.

Collected from two mainly shaded forest ponds, seasonally flooded during the rainy season from October to April, and with high fluctuation level. The ponds are rainwater fed and located in a primary tropical lowland rainforest surrounded by Aguaje palm trees (Figs 11–14). The muddy bottom is covered by broad layers of fallen and rotten leaves and twigs. In the dry season when the surface of the water goes down, a huge, wet area of these leaves and twigs remains. There, and at the edge of the ponds, in small isolated puddles (Fig. 12) of the shallow water zone (less than 10 cm), *Agaporophus julianeae* sp. n. was collected with a dip net, among accumulations of fallen leaves. The species was associated with *Agaporomorphus tambopatensis* Miller, 2005, *Hydrodytes opalinus* (Zimmermann, 1921), *Vatellus grandis* Buquet, 1840, several unidentified species of *Copelatus*, *Hydaticus subfasciatus* Laporte, 1835 (all Dytiscidae), *Tropisternus chalybeus* Laporte, 1840 and several unidentified species of *Helochares* (all Hydrophilidae). In general specimens of *Agaporomorphus julianeae* sp. n. were collected rarely but continuously in the time of observation from May to July (Apenborn 2013).

### Type material examined for comparison

***Agaporomorphus knischi* Zimmermann, 1921**

Fig. 15

**Figures 15–18.**
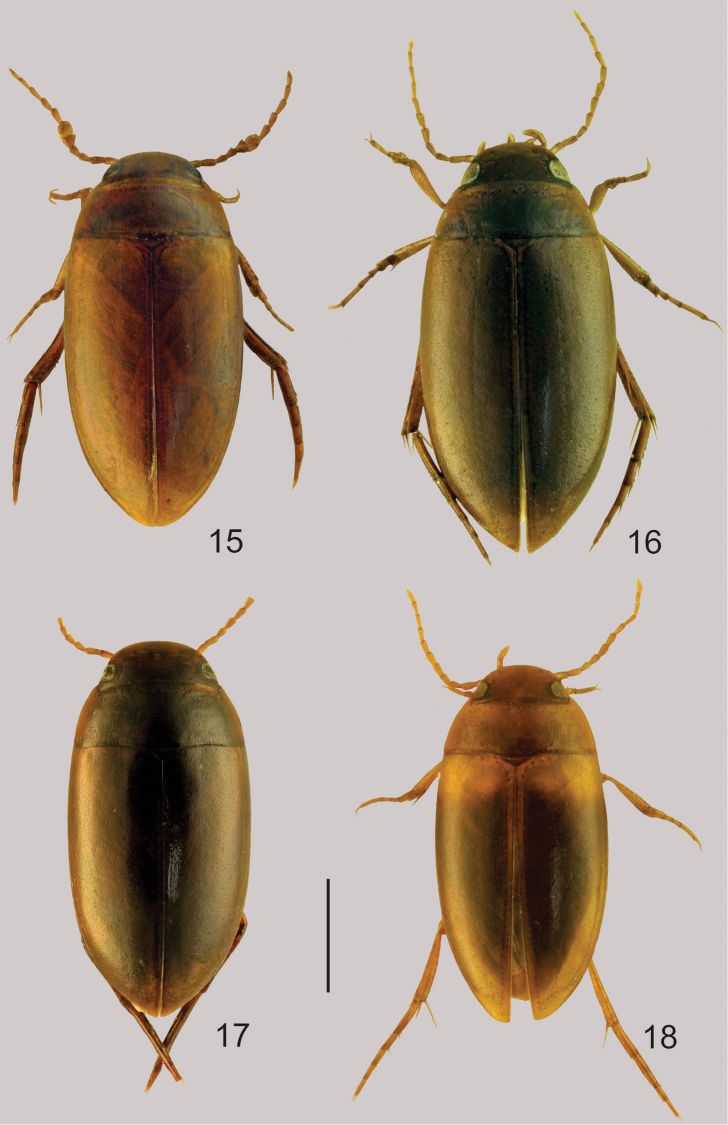
Habitus of *Agaporomorphus knischi*, male, paralectotype (**15**); *Agaporomorphus mecolobus*, male (**16**); *Agaporomorphus pereirai*, male, paratype (**17**) and *Agaporomorphus tambopatensis*, male (**18**). Scale = 1 mm.

**Lectotype.** ♂, “Brasilien”, “Mato Grosso Corumba”, “Type” [blue handwritten label], “Lectotype *Agaporomorphus knischi* Zimmermann, 1921 des. K.B. Miller 2001 [red printed label] (ZSM).

**Paralectotypes.** 12 ♂ ♂, 28 ♀♀, same data as lectotype (ZSM); 10 ♂ ♂, 10 ♀♀, “Corumba [Brazil], Matt [Matto] Grosso”, “W.M. Muche Radebeul Ankauf”, “Staatl. Museum für Tierkunde Dresden” (SMTD).

***Agaporomorphus pereirai* Guignot, 1957**

Fig. 17

**Paratypes.** 3 ♂♂, 5 ♀♀, “Brasilien, Para Cachimbo X 1955 Pereira”, printed genus label, “Paratype” [red printed label], “Museum Paris 1960 Coll. F. Guignot” [light blue printed label] (MNHN).

### Faunistic notes

***Agaporomorphus mecolobus* Miller, 2001**

Fig. 16

*Agaporomorphus mecolobus* Miller, 2001a: 527 (orig. descr.); Miller 2005: 49 (system., catal.); Miller andWheeler 2008: 64 (catal.); Miller 2014: 181 (system., catal.); Nilsson 2015: 46 (catal.).

**Material studied.** 20 ♂♂, 40 ♀♀, “Brasil/Minas Gerais Cordisburgo, Faz. Potinha, XII.1993 [at light] Vaz de Mello leg.” (NMPC, UFMT, ZSM).

**Remarks.** This species was only known after the few type specimens from Sao Paulo (Miller 2001). It is here recorded for the first time for Minas Gerais in Brazil.

***Agaporomorphus tambopatensis* Miller, 2005**

Fig. 18

*Agaporomorphus tambopatensis* Miller, 2005: 52 (orig. descr.); Miller and Wheeler 2008: 64 (system., catal.); Miller 2014: 181 (system., catal.); Nilsson 2015: 46 (catal.).

**Material studied.** 1 ♂ and 1 ♀, “Peru, Dept. Huànuco, ACP Panguana, Rio Yuyapichis, östl. Ort, 9°37'S, 74°56'W, 230 m, 10.05.–25.7.2013, leg. R. Apenborn” (ZSM).

**Remarks.** Described from Madre de Dios, Rio Tambopata in Peru and just known from the type material (Miller 2005). This is the second record of the species in Peru.

***Hydrodytes opalinus* (Zimmermann, 1921)**

Fig. 19

**Figure 19.**
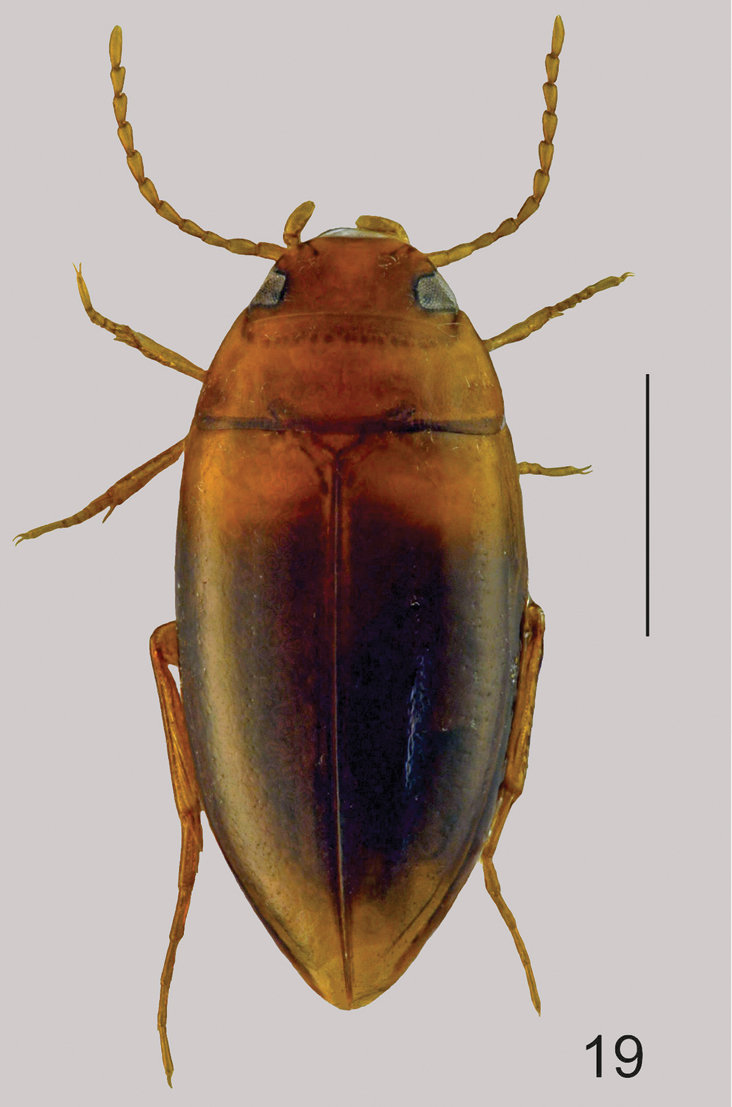
Habitus of *Hydrodytes opalinus*, male (7). Scale = 1 mm.

*Agaporomorphus opalinus* Zimmermann, 1921: 204 (orig. descr.); Guéorguiev 1968: 37 (system., catal.).

*Hydrodytes opalinus* (Zimmermann, 1921); Miller 2001b: 77 (system.); Miller 2002: 6 (system.); Nilsson 2015: 95 (catal.).

**Material studied.** 1 ♂ 3 ♀♀, “Peru, Dept. Huànuco, ACP Panguana, Rio Yuyapichis, östl. Ort, 9°37'S, 74°56'W, 230m, 10.05.–25.7.2013, leg. R. Apenborn” (ZSM).

**Remarks.** Described from Mato Grosso, Brazil and widespread in northern South America (Miller 2002). This is the third record of the species in Peru.

## Supplementary Material

XML Treatment for Agaporomorphus julianeae
